# “*I have nothing more to give*”: Disparities in burnout and the protective role of immigrant status during the COVID-19 pandemic

**DOI:** 10.3389/fpubh.2022.994443

**Published:** 2022-11-16

**Authors:** Gene Chibuchim Otuonye, Nancy Shenoi, Tianshi David Wu, Kalpalatha Guntupalli, Nidal Moukaddam

**Affiliations:** ^1^Department of Critical Care, Baylor College of Medicine, Houston, TX, United States; ^2^Department of Psychiatry, Baylor College of Medicine, Houston, TX, United States

**Keywords:** burnout, COVID-19, immigrants, gender, women

## Abstract

Burnout is an epidemic, with deleterious effects on individuals, patient care, and healthcare systems. The Coronavirus Disease 2019 (COVID-19) pandemic may be exacerbating this problem. We aimed to explore socio-cultural and gender norms that modulate burnout development in physicians during the pandemic and analyze any disparities associated with gender, marital and immigration status and work-life balance. We conducted an online cross-sectional survey of physicians (August–November, 2021): The Maslach Burnout Inventory-Human Services Survey (MBI-HSS) was used to measure burnout, combined with a validated survey assessing work-life balance. Demographic data was obtained for each participant. MBI-HSS subscales were measured, along with work and home related changes due to COVID-19. The association between life changes due to COVID-19 and odds of burnout was estimated by logistic regression. Complementary analysis was performed to determine factors most associated with burnout. 352 respondents were analyzed. There was a high prevalence of burnout. Over half of individuals reported a high degree of emotional exhaustion (EE) (56%). 83% of individuals reported at least one life factor changed due to COVID-19. Home-related life changes due to COVID-19 were associated with 143% higher odds of emotional burnout [adjusted odds ratio (aOR) 2.43; 95% confidence interval (CI) 1.49, 3.98] after covariate adjusted analysis. High EE was most evident when there were three or more life changes, suggesting a cumulative effect. First-generation immigrants, older physicians, and trainees were identified as protective factors. Although female gender was identified as a factor related to EE through forward selection, this was not statistically significant (aOR 1.34; 95% CI 0.80, 2.24). Burnout remains pervasive among physicians. We highlight new risk factors for EE (home-life changes due to COVID-19), and protective factors (first-generation immigrants) not previously explored. Understanding burnout and its disparities allows for improved mitigation strategies, decreasing its deleterious effects.

## Introduction and background

Burnout among health care workers (HCW) is a public health crisis that has reached epidemic proportions (1). Originally described by Freudenberger in 1974 as emotional exhaustion associated with psychological and psychosomatic symptoms, the 11th Revision of the International Classification of Diseases (ICD-11) has since defined it *as an occupational phenomenon resulting from chronic stress in the workplace* that has not been successfully managed (2). It encompasses emotional exhaustion and energy depletion, depersonalization, and reduced professional efficacy (2–4).

Burnout not only has a negative impact on one's self but pervades many aspects of patient care. It has been associated with higher rates of patient morbidity and mortality, hospital length of stay, medical errors, employee attrition, and up to $6.3 billion dollars yearly in US healthcare costs (5, 6). Although efforts to evaluate and mitigate burnout are ongoing (7), HCW burnout rates at best appear to be stagnant (8) and at worst are increasing (4).

The current pandemic has only served to exacerbate the problem globally. A recent publication by The Washington Post/Kaiser Family Foundation Survey Project reported HCW burnout rates as high as 55% in the USA (9). Maunder et al. reported burnout in up to 62% in Canadian HCW (10), and Denning et al. reported burnout symptoms in 67% of HCW in the UK, Poland and Singapore (11). This is likely multifactorial. Increased work hours, fear of personal infection and transmission to family, high patient mortality, unpredictable infection surges, emergence of more virulent and transmissible viral strains, vaccine hesitancy and medical misinformation may be pushing the emotional and psychological wellbeing of HCW to a breaking point.

The pandemic has also brought to the forefront challenges specific to women in medicine. Although women in healthcare have consistently reported higher rates of burnout compared to men over the last few decades, a 2022 national report showed a greater disparity compared to normal: 56% of women reported burnout compared to 41% of men (8). Pre-existing gender-bias and pay inequality in the workplace likely continue to play a role, but factors specific to the pandemic such as increased household responsibilities and coordinating care for “at home children” may exacerbate work-life conflict. In fact, burnout may disproportionately affect HCW not only based on their gender, but also marital and immigration status and family dynamics (4, 8, 12). We hypothesized that immigration status and female gender are protective factors for burnout and sought to identify and analyze these and other possible disparities.

## Methods

After obtaining IRB approval, an anonymous online cross-sectional survey consisting of two main components: the MBI-HSS and a gender-focused questionnaire developed and validated by Raffi et al. (13, 14) was administered through the Qualtrics survey platform to HCW who had worked during the COVID-19 pandemic, including but not limited to those who had direct contact with patients diagnosed with COVID-19.

The MBI-HSS is a psychological assessment tool consisting of 22 items and is the most commonly used method to assess burnout in HCW. Its psychometric properties have been examined and well validated among numerous populations and professions (15, 16). It assesses the 3 main components of burnout scored on a seven-point scale, ranging from 0 (never) to 6 (every day). EE is evaluated over nine questions with a score range of 0–54 (>26 reflected high burnout, 17–26 reflected moderate burnout, and <17 reflected low burnout). Depersonalization is assessed over five questions with a score range of 0–30 points (>12 indicated high burnout, 7–12 reflected moderate burnout, and <7 reflected low burnout). Personal accomplishment is evaluated with eight items with a score range of 0–48 points (<32 reflected high burnout, 32–38 indicated moderate burnout and >38 reflected low burnout).

An established questionnaire by Rafii et al. was also used to characterize aspects of home and work life which have been previously associated with burnout. Evaluated attributes included clinical responsibilities such as time spent seeing patients, household responsibilities such as childcare, and sleep habits, as well as finances, substance/alcohol use and overall sense of wellbeing (14). For each individual attribute, participants reported if there was any change due to COVID-19.

Based on these answers, we determined if a participant had any life attribute, any home life attribute, and any work life attribute changed due to COVID-19. The total number of attributes changed due to COVID-19 was also calculated. The questionnaire also included other variables of interest including: age, female gender, trainee status, immigrant status (first- or second-generation immigrants) and specialty.

Various social media platforms restricted to HCW were used for global recruitment and questionnaire dissemination including: Worldwide Facebook groups: (Physicians Moms Group, Physicians for Patient Protection, The Physician Collective PMG COVID 19 subgroup, COVID-19 Texas Health care Professionals, Novel Coronavirus and COVID-19; Houston Women Physicians, Physician Women in Leadership) and WhatsApp (Pulmonary and Critical Care Fellows and Intensivists at Baylor College of Medicine, Houston, TX, Internal Medicine Residents in various programs in New Jersey, New York, Texas, Jamaica, India). The questionnaire was also sent *via* email (Baylor College of Medicine Alumni group, American University of Beirut and Andhra Medical College, Andhra Pradesh Medical graduates association, American association of Physicians of Indian Origin, Dow Medical school in Pakistan). The survey was only available in English and hence only sent to HCW with English proficiency. There was no randomization, group assignments or follow-up.

### Statistical analysis

The analytic population were all physician respondents who provided complete data, including responses necessary to calculate scores on the MBI-HSS. Life changes due to COVID-19 which were not answered or not applicable for all respondents (such as who cared for children at home) were singly imputed as “no” and were categorized into work-related or home-related changes. Summary statistics were generated.

The association between life changes due to COVID-19 and odds of burnout in each MBI-HSS subscale was estimated by logistic regression. Unadjusted and adjusted estimates were performed. We conducted four separate analyses, parameterizing life changes due to COVID-19 as any life change, any work-related life change, any home-related life change, and a count variable of the total number of life changes. We adjusted *a priori* for age, gender, training status, and medical specialization, presuming these to be important covariates which may confound the relationship between life changes due to COVID-19 and burnout. A two-sided *p* < 0.05 was taken to infer statistical significance.

We next performed a complementary analysis to determine which factors most explained burnout, without forcing life changes due to COVID-19 into the model. Because of the exploratory objectives of the study, we relied on data agnostic techniques to identify these factors and no pre-specified power analysis was conducted. We did backward and forward stepwise techniques with the threshold *p*-value for variable removal or insertion, respectively, of *p* < 0.1, and best subset selection based on the model which produced the lowest Akiake Information Criterion (32148735).

All analyses were performed in Stata 15 (StataCorp; College Station, TX).

## Results

A total of 352 respondents were included in the analysis (Table 1). Mean age was 41 years, and approximately two-thirds were of female gender. Ninety-two (26%) of respondents self-identified as trainees. There was a high prevalence of burnout, with over half of individuals reporting a high degree of EE.

**Table 1:** Demographics and MBI-HSS scales (*n* = 352).

**Factor**	
Age, mean (standard deviation)	40.6 (10.2)
Female gender	234 (66.5%)
Trainee	92 (26.1%)
**Immigrant status**	
Not immigrant	178 (50.6%)
First generation	110 (31.3%)
Second generation	64 (18.2%)
**Specialty**	
Primary care	96 (27.3%)
Critical care	61 (17.3%)
Medical subspecialty	56 (15.9%)
Psychiatry/psychiatry sub	97 (27.6%)
Surgical	42 (11.9%)
Number children, median (interquartile range)	1 (0, 2)
**Household responsibility**	
0–25%	51 (14.5%)
25–50%	106 (30.1%)
50–75%	96 (27.3%)
75–100%	99 (28.1%)
**Hours spent seeing patients**	
< 8	16 (4.5%)
9–16	22 (6.3%)
17–24	36 (10.2%)
25–32	48 (13.6%)
33–40	76 (21.6%)
>40	154 (43.8%)
**MBI-HSS emotional exhaustion**	
Low	67 (19.0%)
Moderate	88 (25.0%)
High	197 (56.0%)
**MBI-HSS depersonalization**	
Low	129 (36.6%)
Moderate	105 (29.8%)
High	118 (33.5%)
**MBI-HSS professional accomplishment**	
Low	107 (30.4%)
Moderate	162 (46.0%)
High	83 (23.6%)

MBI-HSS, Maslach Burnout Inventory-Human Services Survey.

Table 2 summarizes the distribution of life changes that occurred due to COVID-19. Overall, most individuals (83%) reported at least one life factor changed due to COVID-19, with approximately equal proportions of these changes being in factors related to home or work.

**Table 2:** Work and home life changes due to COVID-19.

**Factor**	Value*
Hours spent seeing patients*	154 (43.8%)
Obtaining subspecialty education*	126 (35.8%)
Obtaining CME*	141 (40.1%)
Caregiver for children	56 (15.9%)
Pick up children from school	31 (8.8%)
Care for children if sick	34 (9.7%)
Feeding family	66 (18.8%)
Obtaining groceries	95 (27.0%)
Household responsibility	41 (11.6%)
Employing household help	42 (11.9%)
Use of errand simplifiers	25 (7.1%)
Sleep time	81 (23.0%)
Personal downtime	138 (39.2%)
Any change	291 (82.7%)
Any change at work	243 (69%)
Any change at home	226 (64.2%)
Total changes, median (interquartile range)	2 (1, 4.5)

*Classified as a work change; CME, continuing medical education.

*Value, Respondents who responded in the affirmative.

**Figure 1:**
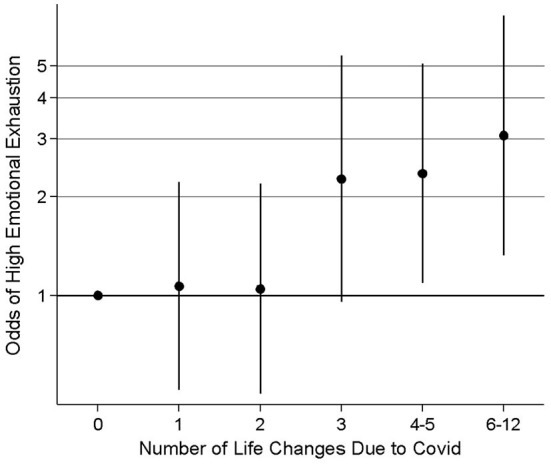
Odds ratios and 95% confidence intervals for emotional burnout associated with number of life changes due to COVID.

Table 3 reports the adjusted relationship between any life change, any work-related life change, and any home-related life change due to COVID-19 with odds of burnout in each of the three MBI-HSS subscales. Unadjusted analysis is available in Table 4. In adjusted analysis, we did not find a statistically significant relationship between any life change due to COVID-19 with burnout in all scales. However, when examining home vs. work-related changes, we found that any home-related life change due to COVID-19 was associated with 143% higher odds of emotional burnout [adjusted odds ratio (aOR) 2.43; 95% confidence interval (CI) 1.49, 3.98]. There were no relationships between work-related life change due to COVID-19 and burnout. When the total number of COVID-19-related life changes were separated into equal sextiles, we found that high EE was most evident when there were three or more life changes, suggesting that the effect is cumulative (Figure 1).

**Table 3:** Adjusted odds ratios (aOR) of burnout associated with life changes due to COVID-19^*^.

**MBI scale**	**Type of life change**		
	**Any life change, aOR (95% CI)**	**Any work-related life change, aOR (95% CI)**	**Any home-related life change, aOR (95% CI)**
High emotional exhaustion	1.57 (0.87, 2.84)	1.24 (0.76, 2.02)	2.43 (1.49, 3.98)
High depersonalization	1.46 (0.77, 2.78)	1.28 (0.76, 2.14)	1.43 (0.85, 2.42)
Low professional achievement	1.09 (0.54, 2.18)	1.34 (0.74, 2.41)	0.83 (0.48, 1.44)

*Adjusted for age, gender, trainee status, and specialty.

**Table 4:** Unadjusted odds ratios (OR) of burnout associated with life changes due to COVID-19.

**MBI-HSS scale**	**Type of life change**		
	**Any life change, OR (95% CI)**	**Any work-related life change, OR (95% CI)**	**Any home-related life change, OR (95% CI)**
High emotional exhaustion	1.77 (1.01, 3.09)	1.31 (0.83, 2.06)	2.55 (1.63, 3.98)
High depersonalization	1.25 (0.69, 2.28)	1.10 (0.68, 1.78)	1.27 (0.80, 2.03)
Low professional achievement	1.04 (0.54, 2.01)	1.33 (0.77, 2.30)	0.75 (0.45, 1.24)

Because we found that life changes due to COVID-19 most impacted the EE dimension of the MBI-HSS, we next investigated if such life changes remained predictive of EE when taken together with all other factors assessed by the survey. All three variable selection methods found that home-related life changes due to COVID-19 retained a substantial relationship with high emotional burnout but work-related life changes did not.

In addition, high emotional burnout was also more likely with a greater number of hours spent seeing patients and greater household responsibility. Conversely, being a first-generation immigrant, being older, and being a trainee were identified as protective factors (Table 5). Both backward selection and best subset selection identified the same factors. Forward selection identified that female gender was related to emotional burnout, but this comparison was not statistically significant (aOR 1.34; 95% CI 0.80, 2.24).

**Table 5:** Factors most associated with emotional burnout^*^.

**Factor**	**Burnout, aOR (95% CI)**
Age (per 1-year increase)	0.96 (0.93, 0.99)
Trainee status	0.33 (0.17, 0.64)
**Immigrant status**	
Not immigrant	Referent
First generation	0.51 (0.30, 0.87)
Second generation	0.91 (0.49, 1.69)
Hours spent seeing patients (per 1-category increase)^a^	1.26 (1.06, 1.49)
Percentage of household responsibility (per 25% increase)	1.31 (1.04, 1.64)
Any home change due to COVID-19	2.61 (1.61, 4.23)

* Identified based on best subset and backward selection methods.

a Categories of hours per week were < 8, 9–16, 17–24, 25–32, 33–40, >40.

## Discussion

Burnout as an entity remains pervasive among HCW. 81% of respondents in this present study self-reported at minimum moderate EE among respondents, with 56% reporting high EE. This correlates with previously published reports examining burnout (1, 9). While burnout is still a subjectively defined phenomenon, the effects of developing burnout can manifest as interpersonal difficulties, increased medical errors, patient morbidity and mortality and ultimately job burnout and attrition (5, 6). Burnout effects are cumulative. Repeated and prolonged EE may precipitate the movement of HCW to different institutions, or to leave medicine altogether. Indeed, The Washington Post/Kaiser Family Foundation Survey Project reports that 29% of HCW have considered leaving the medical field, in part due to burnout (9). Moreover, a recent AAMC report projects a shortfall of up to 124,000 physicians by 2034, with burnout contributing to accelerated rather than delayed retirement (17). There is scant research exploring the socio-cultural and gender norms that may modulate burnout development. In this study we sought to understand the effects, if any, gender and immigrant status had on self-reported burnout in our selected sample, as well the effects of the COVID-19 pandemic on work-life balance and its association with burnout.

In this study, 66.5% of our respondents identified as female. Women have historically reported and continue to report higher rates of burnout than men, and the reasons are not fully understood. A recent 2022 survey of over 13,000 physicians reported burnout rates of 56% in women vs. 41% in men (8). Although not statistically significant, forward selection in this present study also identifies a trend toward increased emotional burnout in women (aOR 1.34; 95% CI 0.80, 2.24). Although women may be more likely to report burnout than men, traditionally gender specific roles of childrearing and household responsibilities may be a contributing factor in working women who had increased home responsibilities due to COVID-19, or who had arranged for help and support prior to the pandemic, but lost it with virus-related restrictions. In fact, in both genders, life changes due to COVID-19 most impacted self-reported EE. Specifically, home changes were associated with 143% higher odds of emotional burnout, with apparent cumulative effects, with increasing likelihood of burnout per additional reported home change. This is particularly significant as the prevailing culture of medicine stigmatizes and discourages help-seeking. Many physicians do not seek care when depressed or anxious and are often expected to function impeccably at work despite personal life changes. The COVID-19 pandemic continues to highlight needed systemic changes in supporting faculty and trainees through personal difficulties as a way to counter the development/worsening of burnout.

To our knowledge, this is the first study of its kind to examine the effect of first and second-generation immigrant status on trainee and non-trainee physician burnout. Immigrant status appears to be protective from EE in our survey. Compared to non-immigrants, first generation immigrants were 49% less likely to report burnout symptoms, and there was a non-statistically significant trend toward less exhaustion among second generation immigrants. The reasons for this are unknown. Although there is a paucity of data regarding burnout among immigrant physicians, West et al. attempted to explore differences in burnout between U.S. and international medical graduates (IMGs) in Internal Medicine residency. They hypothesized that IMGs trainees in US residency programs may be less prone to burnout due to their successful navigation through the complex and highly competitive residency selection process (18). Onge et al. suggested that differences in medical school curricula and emphasis on USMLE scores may play a role (19). We further theorize this may be due to cultural differences as it relates to moral value systems and increased emphasis on labor in many developing countries and under resourced settings. Nevertheless, this finding is in line with the perspective that burnout development is a fairly personal experience with intricate socio-cultural effects that need further investigation. Burnout in immigrant physicians, who constitute 28% of the United States physician workforce, and who train and work in the U.S., appears to be poorly understood, and further study is needed in this population.

Of the three subdivisions of burnout, 81% of respondents reported moderate to high EE compared to 63.3% reporting depersonalization and 30.4% reporting low personal achievement. EE is likely the first manifestation of burnout. We hypothesize that repeated and prolonged EE has a direct temporal relationship to eventual depersonalization and low professional achievement. Future study is needed on this.

We do recognize limitations in this study. Recruitment strategies involved use of various social media platforms for questionnaire dissemination and may have targeted particular groups including but not limited to physicians in Lebanon, India, and Pakistan, acknowledging that various cultures may have alternate views on the responsibilities of men and women at home and at work. Survey completion was also limited to English-speaking participants meaning the sample was not globally representative. Moreover, there was no randomization within the study of participants which potentially affects the generalizability of results.

The survey design also inquired less about work-related changes than home-related changes, taking into account only hours spent working and time spent obtaining CME and subspecialty education. Future assessments of burnout may need to assess other work-related aspects of work-life balance in order to have a more comprehensive understanding of physician burnout. Most studies about physician burnout identify key workplace factors such as dealing with patient death and illness, a loss of autonomy at work, decreased sense of control over work due to administrative requirements, clerical stressors related to utilization of electronic health record usage, and frequent call duties (5). It is possible that such factors may compromise the delivery of health care services and lead to increased rates of medical errors and decreased job satisfaction. The implications of work-related changes could be far-reaching and should be a topic of future study.

Another consideration in this study was that a significant sector of the study population was excluded due to incomplete survey answers, particularly to questions about gender expectation, a core area our group was interested in studying.

## Conclusion

Burnout remains a global epidemic affecting the healthcare sector with significant impact on medical workers' individual lives and on patient care. The COVID-19 pandemic, still ongoing after 2 years and with no clear signs of remitting, has had unprecedented implications for HCW work-life balance. This worldwide cross-sectional survey brings to light unique risk factors for emotional exhaustion and burnout during the pandemic as well as unique protective factors. First and second-generation immigrant status, a factor not known to be previously explored in this manner, appears to be protective from EE in our survey. Only by understanding high risk workers who may develop high levels of EE, depersonalization and decreased personal accomplishment, can we enact effective mitigation strategies to prevent end-stage burnout and subsequent exodus from the field of medicine.

## Data availability statement

The raw data supporting the conclusions of this article will be made available by the authors, without undue reservation.

## Ethics statement

The studies involving human participants were reviewed and approved by the Institutional Review Board for Human Subject Research for Baylor College of Medicine and Affiliated Hospitals (BCM IRB). The patients/participants provided their written informed consent to participate in this study.

## Author contributions

GO: literature review, chart review, data and results analysis, and grammatical composition. NS: data review, discussion, and conclusion drafts. TW: statistical analysis. NM and KG: hypothesis and methods outline, questionnaire formulation, IRB applications, overall guidance, and review of project including suggestions for discussion. All authors contributed to the article and approved the submitted version.

## Conflict of interest

The authors declare that the research was conducted in the absence of any commercial or financial relationships that could be construed as a potential conflict of interest.

## Publisher's note

All claims expressed in this article are solely those of the authors and do not necessarily represent those of their affiliated organizations, or those of the publisher, the editors and the reviewers. Any product that may be evaluated in this article, or claim that may be made by its manufacturer, is not guaranteed or endorsed by the publisher.
